# Prenatal alcohol exposure is a leading cause of interneuronopathy in humans

**DOI:** 10.1186/s40478-020-01089-z

**Published:** 2020-11-30

**Authors:** Florent Marguet, Gaëlle Friocourt, Mélanie Brosolo, Fanny Sauvestre, Pascale Marcorelles, Céline Lesueur, Stéphane Marret, Bruno J. Gonzalez, Annie Laquerrière

**Affiliations:** 1 Department of Pathology, Normandie Univ, UNIROUEN, INSERM U1245 and Rouen University Hospital, Normandy Centre for Genomic and Personalized Medicine, 76000 Rouen, France; 2 Faculté de Médecine et des Sciences de la Santé, Inserm UMR1078, Université de Bretagne Occidentale, Brest, France; 3 Etablissement Français du Sang (EFS), Bretagne, France; 4 Laboratoire de Génétique Moléculaire, CHRU Brest, Hôpital Morvan, Brest, France; 5 Normandie Univ, UNIROUEN, Normandy Centre for Genomic and Personalized Medicine, INSERM U1245, 76000 Rouen, France; 6 Department of Pathology, Bordeaux University Hospital, Bordeaux, France; 7 Department of Pathology, Brest University Hospital, Brest, France; 8 Laboratory of Neurosciences of Brest, Faculté de Médecine et des Sciences de la Santé, Université de Bretagne Occidentale, Brest, France; 9 Department of Neonatal Paediatrics and Intensive Care, Normandie Univ, UNIROUEN, INSERM U1245 and Rouen University Hospital, Normandy Centre for Genomic and Personalized Medicine, 76000 Rouen, France

**Keywords:** GABAergic system defects, Human foetal/neonatal brain, Foetal alcohol syndrome, Immunohistochemistry, Vascular interneuron migration

## Abstract

Alcohol affects multiple neurotransmitter systems, notably the GABAergic system and has been recognised for a long time as particularly damaging during critical stages of brain development. Nevertheless, data from the literature are most often derived from animal or in vitro models. In order to study the production, migration and cortical density disturbances of GABAergic interneurons upon prenatal alcohol exposure, we performed immunohistochemical studies by means of the proliferation marker Ki67, GABA and calretinin antibodies in the frontal cortical plate of 17 foetal and infant brains antenatally exposed to alcohol, aged 15 weeks’ gestation to 22 postnatal months and in the ganglionic eminences and the subventricular zone of the dorsal telencephalon until their regression, i.e., 34 weeks’ gestation. Results were compared with those obtained in 17 control brains aged 14 weeks of gestation to 35 postnatal months. We also focused on interneuron vascular migration along the cortical microvessels by confocal microscopy with double immunolabellings using Glut1, GABA and calretinin. Semi-quantitative and quantitative analyses of GABAergic and calretininergic interneuron density allowed us to identify an insufficient and delayed production of GABAergic interneurons in the ganglionic eminences during the two first trimesters of the pregnancy and a delayed incorporation into the laminar structures of the frontal cortex. Moreover, a mispositioning of GABAergic and calretininergic interneurons persisted throughout the foetal life, these cells being located in the deep layers instead of the superficial layers II and III. Moreover, vascular migration of calretininergic interneurons within the cortical plate was impaired, as reflected by low numbers of interneurons observed close to the cortical perforating vessel walls that may in part explain their abnormal intracortical distribution. Our results are globally concordant with those previously obtained in mouse models, in which alcohol has been shown to induce an interneuronopathy by affecting interneuron density and positioning within the cortical plate, and which could account for the neurological disabilities observed in children with foetal alcohol disorder spectrum.

## Introduction

The mammalian neocortex contains two major classes of neurons, projection and local circuit neurons: projection neurons which contain the excitatory neurotransmitter glutamate, and local circuit neurons which are mainly inhibitory and contain the neurotransmitter GABA (γ-aminobutyric acid). GABAergic interneurons (INs) which represent between 25 and 30% of cortical neurons [26, 66] derive from progenitor cells located in the ventricular (VZ) and subventricular zones (SVZ) of the ventral telencephalon including median, lateral and caudal ganglionic eminences (MGE, LGE and CGE), as well as from the preoptic, preoptic-hypothalamic and septal areas. In humans, their origin has been a matter of debate for a long time, and over the last 20 years some authors have reported that contrary to rodents, a proportion of INs in humans could arise from the dorsal telencephalon [24, 32, 44, 69]. But it is now well admitted that the majority of INs in primates including humans originate in the ganglionic eminences (GE) [2, 21, 36]. GABAergic IN specification within the GE is linked to the expression of transcription factors encoded by a set of regulatory genes such as *Dlx1* (Distal less homeobox gene), *Dlx2*, *Ascl1* (Achaete-scute family bHLH transcription factor 1) formerly known as *Mash1*, *Gsx1* and *Gsx2* (Genetic-Screened Homeobox 1 and 2) [34, 61]. The emergence of morphological and functional IN diversity is related to the spatial and temporal specification of progenitor cells according to additional transcriptional programs that are either intrinsically encoded or activated by interactions with the local microenvironment [33]. Two lineages of neocortical GABAergic INs exist. The first expresses Dlx1/2 transcription factors, and represents 65% of neocortical GABAergic neurons, originating from Ascl1 expressing progenitors. The second lineage, which expresses Dlx1/2 but not Ascl1, forms around 35% of GABAergic INs [3, 45]. In rodents, *Dlx1/2, Nkx2.1, Lhx6, Lhx7* and *ARX* participate in the control of GABAergic IN production and migration [16].

GABAergic INs arising from the MGE and CGE migrate following tangential migratory routes parallel to the brain surface, then through the marginal zone to enter the cortex at early stages, through the intermediate/subventricular zone and subplate at later stages, and finally migrate radially to their final position in the appropriate cortical layers. Migration has been shown to be regulated by motogenic factors [40], by non-classical microtubule associated proteins [5] and by either chemoattractive or repulsive molecules [37–39, 63]. During the migration process, INs progressively acquire their characteristic morphology as well as their molecular, biochemical and synaptic properties. IN incorporation into the laminar structures of the cortical plate has been shown to be controlled, at least partly, by pyramidal neurons [68].

More than 20 different types of inhibitory INs have been described in the murine neocortex and hippocampus [58] and are divided into several classes and subclasses according to their morphology, their potential transcriptional similarities and to selective marker expression. Three major classes are recognized depending on parvalbumin, somatostatin and serotonin receptor 3a (Htr3a) expression [33]. INs expressing Htr3a comprise bipolar INs co-expressing calretinin (CR), as well as single bouquet cells, interstitial or multipolar neurons and basket cells which co-express cholecystikinin, vasointestinal peptide, reelin, or neuropeptide Y [reviewed by 33].

The main functions of GABA-mediated neurotransmission consist in synchronizing neuronal networks by modulating cortical output, controlling neuronal excitability and information processing as well as neuronal plasticity [10]. In the developing rodent brain, this neurotransmitter is excitatory which could be explained by a higher intracellular concentration in chloride ions at early developmental stages [9]. The switch from excitatory to inhibitory function occurs between the second and seventh postnatal day, equivalent to the 3rd trimester of gestation in humans [8]. In the perinatal human neocortex and hippocampus, this excitatory action could play an important role in controlling several processes including cell proliferation in the germinative zones, post-mitotic neuroblast migration and cell differentiation, and may transiently regulate neuronal growth and dendritic maturation before synaptogenesis [10, 35, 67].

Aberrant development and function of GABAergic systems in humans have been implicated in various pathologies such as XLAG-syndrome (MIM# 300004) [11], epilepsy linked to focal cortical dysgeneses [18] or prenatal white matter injury [51]. GABA disturbances have also been implicated in a number of autism spectrum disorders of known origin such as Fragile X and Rett syndromes [12] as well as in schizophrenia [46]. Recently, Smiley et al. also identified a selective reduction in GABA INs using a murine model of Foetal Alcohol Spectrum disorder (FASD) [57]. In utero alcohol exposure has damaging effects on brain development and is known to be responsible for severe neurodevelopmental disabilities leading to long-term neurobehavioural deficits [4, 7]. The most severe end of the spectrum is Foetal Alcohol Syndrome (FAS) which is characterized by intra-uterine and postnatal growth retardation, typical craniofacial dysmorphism, central nervous system (CNS) structural anomalies as well as behavioural and neurocognitive deficits [27, 50]. For these reasons, most studies have focused on neurons and glial cells to study the mechanisms involved in the deleterious effects of alcohol on brain development [28, 64]. Other studies have reported that in utero alcohol exposure affects brain angiogenesis in particular cortical microvessel organization in both mouse and human foetuses [17, 25, 29, 62]. It has also been reported that some nerve cell types, notably GABAergic INs require brain microvessels to properly migrate toward the cortical plate [35, 65].

From these reports, it could be speculated that developmental abnormalities of GABAergic systems including generation, migration and cortical distribution may account for neurocognitive and behavioural disabilities as well as epilepsy observed in FASD children. These events can be studied using immunohistochemical techniques based on the fact that a substantial proportion of GABAergic INs co-express calcium binding proteins (CaBP), in particular CR which acts as a buffer for modulating intracellular calcium transients [14]. The recent advances in understanding the physiopathology of FASD led us to perform a detailed immunohistochemical study using GABA and CR antibodies in the foetal and postnatal human forebrain of FASD and controls from 14 WG to three years of age. The goals of the present study were first to provide an ontogenetic study of GABAergic IN population and of its calretininergic subpopulation in the germinal zones during normal development by comparison with subjects antenatally exposed to alcohol; second to study their density and repartition in the cortical plate and to compare the spatio-temporal distribution of GABAergic systems; third to search for possible IN positioning abnormalities and fourth whether this mispositioning could be due to their inability to migrate along the intracortical microvessels as vascular migration anomalies have previously been identified in FASD rodent models.

## Patients and methods

### Patients

The brains used in this study belong to the collection which has been declared to the French Ministry of Health (collection number DC-2015-2468, cession number AC-2015-2467, located in A. Laquerrière’s Pathology Laboratory, Rouen University hospital, France). For all selected cases, the parents gave their consent for neuropathological studies of the foetuses or infants following autopsy performed in agreement with the local ethic committee and in accordance with the French law.

Seventeen foetal and postnatal age-matched control brains ranging from 14 WG to 35 postnatal months were selected for the present study, and whose main clinical and morphological characteristics are presented in Table 1. In 7 out of the 17 cases, a medical termination of the pregnancy (TOP) was achieved for pathologies/malformations other than cerebral. Two out of the 17 cases were in utero foetal death (IUFD) with no found cause and 6 out of the 17 cases were perpartum or immediate postpartum death with no found reason or with a cause other than cerebral. The two children aged 3 months and 35 months died of prone position sleep and anaphylactic shock, respectively. In all cases, the brain was macroscopically and microscopically free of detectable abnormalities. Patients who had been suspected of central nervous system anomalies or of dying from neurological causes were systematically excluded.

**Table 1:** Gestational age/postnatal age and cause of death of selected control cases

Case number	Term	Cerebral maturation*	TOP	Cause of death
1	15 WG	14 WG	Yes	Atrioventricular canal
2	16WG	16WG	Yes	Isolated sacral myelomeningocele
3	22 WG	20 WG	No	IUFD
4	22 WG	22 WG	Yes	Obstructive uropathy
5	24 WG	24 WG	No	IUFD
6	26 WG	26 WG	Yes	Hereditary bilateral microphtalmia
7	28 WG	28 WG	Yes	Severe distal arthrogryposis
8	30 WG	30 WG	No	Cord prolapse
9	32 WG	32 WG	Yes	Complex cardiac malformation
10	34 WG	34 WG	Yes	Suspected vermis hypoplasia (Not confirmed)
11	35 WG	35 WG	No	Non immune fetal hydrops Caesarean section
12	36 WG	36 WG	No	Bilateral renal agenesis Fallot tetralogy Immediate postpartum death
13	36 WG	36 WG	No	Dilated cardiomyopathy Dead at day 2
14	37 WG	37 WG	No	Normal pregnancy Death despite resuscitation No autopsy and placental abnormalities
15	39 WG	39 WG	No	Perpartum in utero death Placental membrane praevia vessel rupture
16	3 months PN	3 months PN	No	Prone position sleep
17	2 years and 11 months	3 years	No	Anaphylactic shock

*IUFD* in utero fetal death, *PN* post-natal, *TOP* Medical termination of pregnancy, *WG* weeks’gestation

*According to the morphometric criteria of Guihard-Costa and Larroche [19]

Seventeen foetal and postnatal FASD brains ranging from 15 WG to 22 postnatal months were also included in this study. Detailed clinical and morphological characteristics are presented in Table 2. Causes of death were IUFD in 6 cases, TOP for foetal malformations in 8 cases, post-natal early death in one case and sudden infant death syndrome in the two post-natal cases.

**Table 2:** Main clinical and morphological characteristics of antenatal alcohol exposed fetuses and infants

Case number	WG	Cause of death	Body weight	Cranio-facial dysmorphism	Brain weight	Visceral anomalies	CNS anomalies	Maternal alcohol intake	Maternal Co-morbidity
1	15	TOP	25^th^ percentile	Indistinct philtrum Low set posteriorly rotated ears Microretrognathism	50^th^ percentile 15.8 g	Anterior coelosomia	No	Chronic alcohol intake*	Heroin Methadone
2	20	TOP Maternal distress	10^th^ percentile	Midface hypoplasia, short nose, flat face	25^th^ percentile 39.34 g	No	No	Chronic alcohol intake and Binge drinking* (ND)	HIV, Hepatitis C Multi-drug addiction
3	22	TOP Chiari malformation	<3^rd^ percentile IUGR	FAS craniofacial dysmorphism	≪3^rd^ percentile 45.65 g	Renal hydronephrosis	Microcephaly Arnold Chiari II Myelomeningocele	Chronic alcohol intake**	Psychotic Disorder Valproate
4	22	TOP Maternal distress	3^rd^ percentile IUGR	Indistinct philtrum Posteriorly rotated ears Microretrognathism	50^th^ percentile 63.5 g	No	No	Chronic alcohol intake (262 g per day) **	Psychotic Disorder psychotrops Increased MGV
5	22	TOP Maternal distress	50^th^ percentile	Indistinct philtrum Microretrognathism	50^th^ percentile 63.21 g	Unilateral pelvicaliceal dilatation	No	Daily chronic alcohol intake*	Multi-drug addiction Valproate (epilepsy)
6	24	TOP Amnion band sequence	NA	FAS craniofacial dysmorphism	50^th^ percentile 75 g	NA	Micropolygyria Migration anomalies Vermis hypoplasia cerebellar hemisphere necrosis	Chronic alcohol intake**	Cocaine
7	24	TOP septal agenesis	50^th^ percentile	FAS dysmorphism	50^th^ percentile 100.3 g	No	Arhinencephaly	Chronic alcohol intake**	Cannabis addiction
8	26	Spontaneous abortion	50^th^ percentile	Indistinct philtrum Posteriorly rotated ears Microretrognathism	50^th^ percentile 133.6 g	Unilateral pelvic dilatation	No	Chronic alcohol intake**	ND
9	27	Preeclampsia Death at D10	10^th^ percentile	FAS craniofacial dysmorphism	3^rd^ percentile 109 g	No	Microcephaly Massive cerebellar haemorraghe	Alcohol intake**	ND
10	29	IUFD cardiopathy	5^th^ percentile	Anteversed nostrils and pointed nose Ear anomalies Indistinct philtrum Retrognathism	5th percentile 178 g	Tetralogy of Fallot	No	Daily chronic alcohol intake* (ND)	Increased MGV and GGT
11	30	IUFD Abruptio placentae	10^th^ percentile	No	50^th^ percentile 211 g	No	Bilateral intraventricular haemorrhage	Daily chronic alcohol intake*	Cannabis addiction Treated hypothyroidism
12	31	IUFD Preeclampsia	3^rd^ percentile IUGR	Ear anomalies Retrognathism	3^rd^ percentile 197.05 g	No	No	Daily chronic alcohol intake* (ND)	Increased MGV and GGT
13	31	TOP Septal agenesis	50^th^ percentile	FAS craniofacial dysmorphism	50th percentile 234 g	No	Septal agenesis	Chronic and Binge drinking*	Cannabis
14	33	IUFD Acute alcohol intoxication (maternal distress)	50^th^ percentile	No	50th percentile 348.15 g	Amniotic fluid inhalation	Diffuse astrogliosis	Chronic and Binge drinking* (4.98 g/L)	Multi-drug addiction Increased MGV and GGT First pregnancy: IUFD at 33 WG One child alive with FAS.
15	37	IUFD	25^th^ percentile	Indistinct philtrum Microretrognathism	<3^rd^ percentile 248 g	No	Neuron heterotopia Microcephaly	Chronic and Binge drinking**	Heroin addiction
16	3 PN months	SIDS	5^th^ percentile	FAS cranio-facial dysmorphism	50th percentile 633 g	No	Brain oedema	Chronic alcohol intake* (ND)	Cannabis addiction
17	22 PN months	SIDS	50^th^ percentile	FAS cranio-facial dysmorphism	ND	Isolated pulmonary oedema	No	Daily chronic alcohol intake* (ND)	ND

*CNS* central nervous system, *GGT* gamma-glutamyl transferase, *IUFD* in utero fetal death, *IUGR* intra uterine growth retardation; MGV mean globular volume, *MTP* medical termination of the pregnancy, *ND* not determined, *PN* post-natal, *SIDS* sudden infant death syndrome, *WG* weeks’ gestation

*Maternal self report; **Suspected

### Methods

In each control and FASD patient, a complete autopsy had been performed with the written consent of the parents and according to standardized protocols including X-rays, photographs, macroscopical and microscopical examination of all viscerae and brain [22]. Developmental age was evaluated by means of organ weights [20, 53], skeletal measurements and by the histological maturational stages of the different viscera.

#### Neuropathological studies

Brain growth was evaluated according to the criteria of Guihard-Costa and Larroche [19]. Macroscopic evaluation of brain maturation, in particular gyration, was performed according to the atlas of Feess-Higgins and Larroche [15]. After fixation in a zinc-10% formalin buffer solution for at least 1 month, several brain sections were obtained from frontal, temporal, parietal, cingular and occipital cortices including the calcarine fissure, as well as limbic structures, and basal ganglia including thalamus, striatum, pallidum and their related structures. Multiple seven micrometer paraffin embedded sections were stained using Haematoxylin–Eosin and Cresyl Violet, which made it possible to confirm the absence of cerebral lesions. The morphology of all different brain structures studied was consistent with the patients’ ages.

#### Immunohistochemistry

Immunohistochemical analyses of GABAergic systems were carried out on 6-micrometer sections obtained from paraffin-embedded, according to standardized protocols using antisera directed against GABA (Rabbit polyclonal, diluted 1/100; Thermofisher Scientific, F67403 Illkirch Cedex, france); CR (rabbit monoclonal, diluted 1/100; Life Technology Invitrogen, Villebon sur Yvette, France); Glut1 (Rabbit polyclonal, diluted 1/100; Dakopatts, Trappes, France) and Ki67 (mouse monoclonal, diluted 1/100; Dakoppatts). Immunohistochemical procedures included a microwave pre-treatment protocol to aid antigen retrieval (pre-treatment CC1 kit, Ventana Medical Systems Inc, Tucson AZ). Incubations were performed for 32 min at room temperature using the Ventana Benchmark XT system. After incubation, slides were processed by means of the Ultraview Universal DAB detection kit (Ventana). Semi-quantitative analysis of GABA and CR INs in the germinal and intermediate zones as well as in the different layers of the frontal cortex was evaluated as follows: 0: no cell labelled; +: very few cells labelled (less than 10% of cells); ++: some cells labelled (10-25%); +++: a relatively high proportion of cells labelled (25-50%) and ++++ : most of the cells of the structure are strongly labelled (> 50%).

#### Quantitative analysis of GABAergic cortical interneuron number and density

Quantitative analyses of GABA were carried out in the frontal cortical plate in 15 cases including 8 FASD aged 26 WG to 3 post-natal months. Quantitative analyses of CR were performed in the same cortical area in 11 cases including 5 FASD aged 20, 24, 30, 33 and 37 WG. For measurements of GABA and/or CR positive cell density in the cortical plate, images were acquired and saved in TIFF format using a Leica DMI 6000B microscope. Images were subsequently opened in Mercator software and regions of interest (ROIs) were drawn. Afterwards, a counting frame was defined within the ROI and a threshold was set in order to differentiate immunoreactive cells from the background. By a segmentation process, the computer calculated the number and the cumulated area of objects corresponding to immunoreactive cell somata within the ROI, yielding cell number and cell density per 10^4^ µm^2^.

#### Confocal analyses of interneuron migration in the cortical plate

For confocal analyses, double immunolabellings were performed using GABA or CR and Glut1 antibodies in the frontal cortex at early stages from 14 to 20 WG, and at late stages from 33 to 37 WG. Fluorescent-conjugated antibodies Alexafluor-488 and -592 were obtained from Molecular Probes (Eurogene, Or, USA). After 3 gentle washes in a phosphate buffer solution, coverslips were mounted in DAPI-containing Vectashield (Vector laboratories, Cambridgeshire, UK) and images were acquired with the Leica laser scanning confocal microscope TCS SP2 AOBS (Leica Microsystems, Wetzlar, Germany).

### Statistical analyses

Statistical analyses were performed using the GraphPad Prism software. Chi square test was used to compare the intrinsic distribution of CR-positive cells in cortical plate between FASD and control brains at 24 and 30 WG.

## Results

### FAS patients’ clinical and morphological characteristics

Among the 17 cases exposed to alcohol, 3 (18%) had intra uterine growth retardation (IUGR). All but 2 cases (88%) had cranio-facial dysmorphism which was characteristic of FAS in 7 of the cases (47%), associating an elongated and narrow forehead, short palpebral fissures, hypo- or hypertelorism and epicanthus, midface hypoplasia, a short nose with a broad nasal bridge and anteverted nostrils, indiscernible nasolabial folds, smooth and prominent philtrum, thin vermillion border and micrognathia. Five cases (29%) had microcephaly (brain weight below the 3^rd^ percentile). In 9 cases (53%) other CNS anomalies were identified: myelomeningocele with Chiari II malformation, arhinencephaly, migration abnormalities consisting either in polymicrogyria or neuronal heterotopias and vermis hypoplasia. Clastic lesions consisted of massive cerebellar haemorrhage, cerebellar hemispheric necrosis, bilateral intraventricular haemorrhage, septal agenesis, diffuse astrogliosis and brain oedema. Five cases (29%) had associated visceral malformations: anterior coelosomia, renal hydronephrosis, unilateral pelvic dilatation and tetralogy of Fallot. Amniotic fluid inhalation and isolated pulmonary oedema were diagnosed in 2 cases.

In 10 cases, alcohol intake was self-reported by the mother (59%), consisting in daily chronic alcohol intake, associated with binge drinking in 3 cases. In the other 7 cases (41%), maternal alcohol intake was clinically suspected according to the criteria established by Riley et al. or reported by the family environment [50]. Twelve mothers (71%) had co-morbidities consisting of multidrug addiction or antiepileptic drugs or psychotic traits. Among the 7 cases in which alcohol intake was suspected, 1 case had FAS craniofacial dysmorphism, IUGR and microcephaly with additional CNS anomalies; 1 case had FAS cranio-facial dysmorphism and microcephaly and 1 case had cranio-facial dysmorphism resembling the FAS dysmorphism associated with IUGR. Among these 7 cases, 2 had CNS anomalies with FAS craniofacial dysmorphism; 1 of which had craniofacial dysmorphism resembling the FAS (maternal alcohol intake reported by close relatives). One case had FAS craniofacial dysmorphism, microcephaly and CNS anomalies and 1 case had microcephaly with CNS anomalies and craniofacial dysmorphism reminiscent of FAS, although not characteristic.

### Semi-quantitative and quantitative analyses of GABA, Calretinin and Ki67 immunohistochemistry

#### GABA immunohistochemistry

**Fig. 1:**
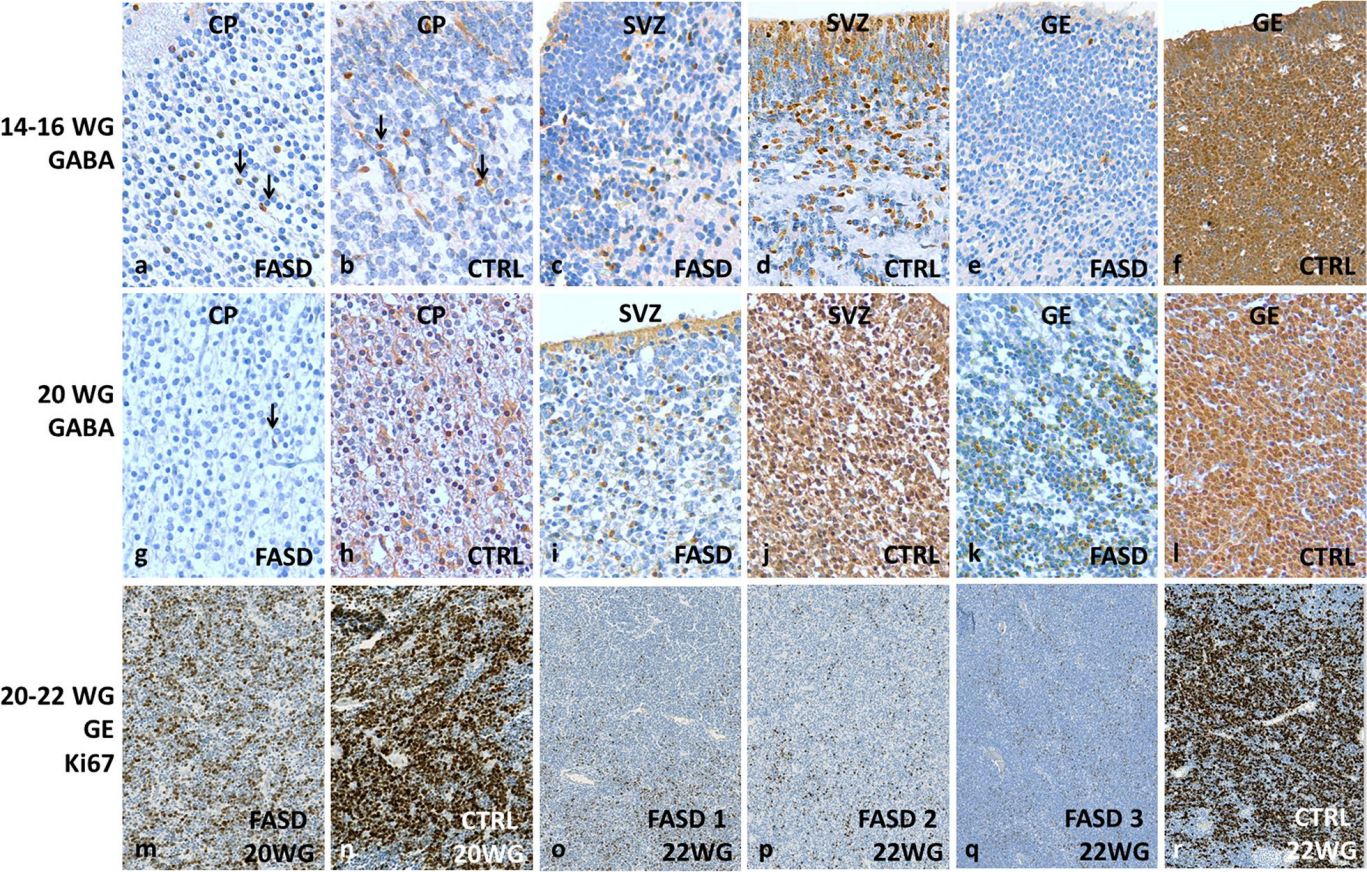
GABA and Ki67 immunohistochemistry in the brains of FASD and controls at early developmental stages and at mid-gestation. Similar GABA interneuron density in cortical plate of FASD and controls at 14-16 WG which contains bipolar INs migrating along the perforating microvessels as indicated by arrows (**a, b**) [OM × 200], contrasting with the low number of GABA interneurons in the VZ/SVZ (**c, d**) [OM x200], and in the GE in FASD compared to control brains (**e, f**) [OM x100)]. At 20 WG, GABA interneurons were scarce in the cortical plate of FASD compared to control (**g, h**) [OM x 200], in the VZ/SVZ (**i, j**) [OM x 200] and in the GE (**k, l**) [OM x 200]. Low proliferative activity revealed by Ki67 immunohistochemistry in the GE of the FASD brains aged 20 and 22 WG compared to control brains (**m-r**) [OM x 100]. CP: cortical plate; CTRL: control cases; GE: ganglionic eminence; FASD: foetal alcohol spectrum disorder; OM: original magnification; SVZ: cortical subventricular zone

During the early foetal period (14-16 WG), cortical GABAergic IN density was only slightly diminished in FASD in comparison with control cortical plates through which they were migrating along the perforating cortical microvessels from the transient subpial granular cell layer (SGL) (Fig. 1a, b). However, GABA-immunoreactive INs were drastically reduced in the cortical VZ/SVZ (Fig. 1c, d) and the GE of FASD (Fig. 1e, f), reflecting a delayed migration toward the cortical SVZ from the GE where they appeared to be insufficiently generated. At mid-gestation (20, 22 and 24 WG), a developmental stage in which there is normally a large production of cortical interneurons, very few GABA-immunoreactive INs were observed, dispersed in the cortical plate (Fig. 1g, h), in the cortical SVZ (Fig. 1i, j), as well as in the GE (Fig. 1k, l), thus confirming our observations at earlier stages in FASD brains. Moreover, similar abnormalities were noted in the 3 FASD brains aged 22 WG, and in the 2 FASD brains aged 24 WG. The low density of GABAergic INs was correlated with very low Ki67 proliferation indices in the GE in comparison with control brains (Fig. 1m–r), confirming a defect in the generation of neurons at these stages. As projection neuron production in the cortical VZ/SVZ ceases from 26 WG and there is no more enrichment in glutamatergic neurons in the cortex, GABAergic IN density tended to be similar to that observed in control brains, without any overt increase in the GE up to 34 WG, a developmental stage when GE physiologically regress. But some cells remained immunolabelled in the GE and the cortical SVZ, likely corresponding to IN intermediate progenitors.

**Fig. 2:**
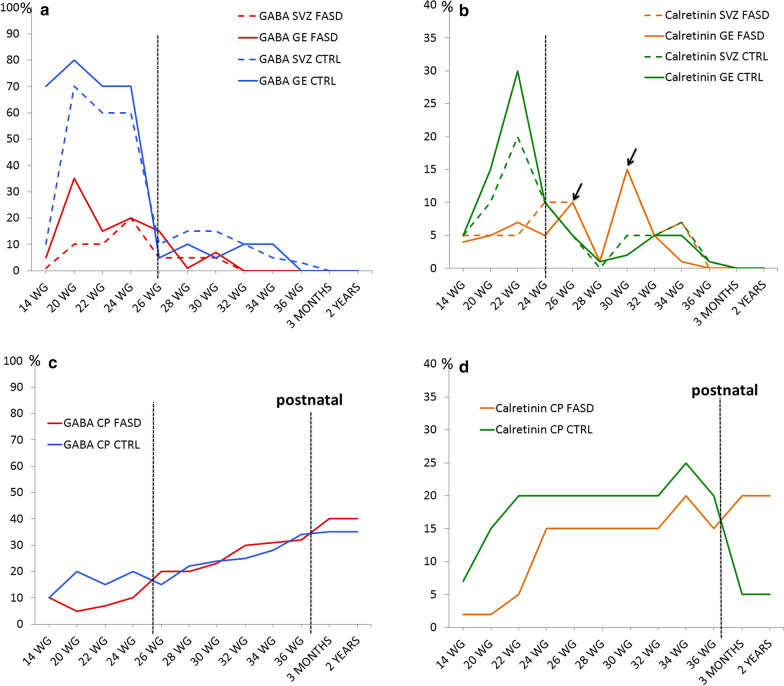
Schematic representation of GABA and CR containing interneurons in the GE, VZ/SVZ and in the cortical plate of FASD and control brains during foetal and post-natal life. **(a)** Evolution of GABA interneuron density in the GE and VZ/SVZ obtained from semi-quantitative immunohistochemical evaluation. The vertical dotted line indicates the stage from which no significant differences between FASD and controls were observed (26 WG). Between 20 and 26 WG, an intense generation of GABA-positive cells was observed in control brains. (**b)** Evolution of CR interneuron density in the GE and VZ/SVZ obtained from semi-quantitative immunohistochemical evaluation. The vertical dotted line indicates the stage at which no significant differences between FASD and controls were observed (24 WG). Between 20 and 24 WG, an intense generation of CR-positive cells was observed in control brains and after 24 WG, two smaller peaks of production were observed in FASD brains indicating a production delay of two months. **(c)** Evolution of cortical density in GABA interneurons. Before 26 WG, cortical interneuron density was lower in FASD, from 26 WG to birth, no major differences were observed between FASD and controls. After birth, cortical density was slightly increased in FASD. **(d)** Evolution of cortical density in CR interneurons. Before birth, the density of CR interneurons was constantly lower in FASD cortices with a reverse pattern after birth, indicating a delayed settling of CR interneurons in the cortex of FASD

Semi-quantitative analyses concerning the developmental patterns of GABA immunoreactivities in the GE, cortical VZ/SVZ and frontal cortical plate of FASD and controls are detailed in Additional file 1 and summarized in Fig. 2. Detailed results obtained from quantitative analyses are presented in Additional file 2.

#### Calretinin immunohistochemistry

In both FASD and control layer I, Cajal Retzius cells were positive for CR antisera, along with the superficial tangential fibre network. This immunoreactivity was observed throughout foetal life and decreased from 38 WG, with a loss of positivity at 3 post-natal months. Additional small rounded neurons located in the molecular layer corresponding to pioneer neurons were also observed 14 WG onwards. In the intermediate zone, dispersed migrating CR-positive INs were observed at all developmental stages until birth and just below the cortical plate, dispersed CR-positive cells corresponding to subplate interneurons were noted until birth.

**Fig. 3:**
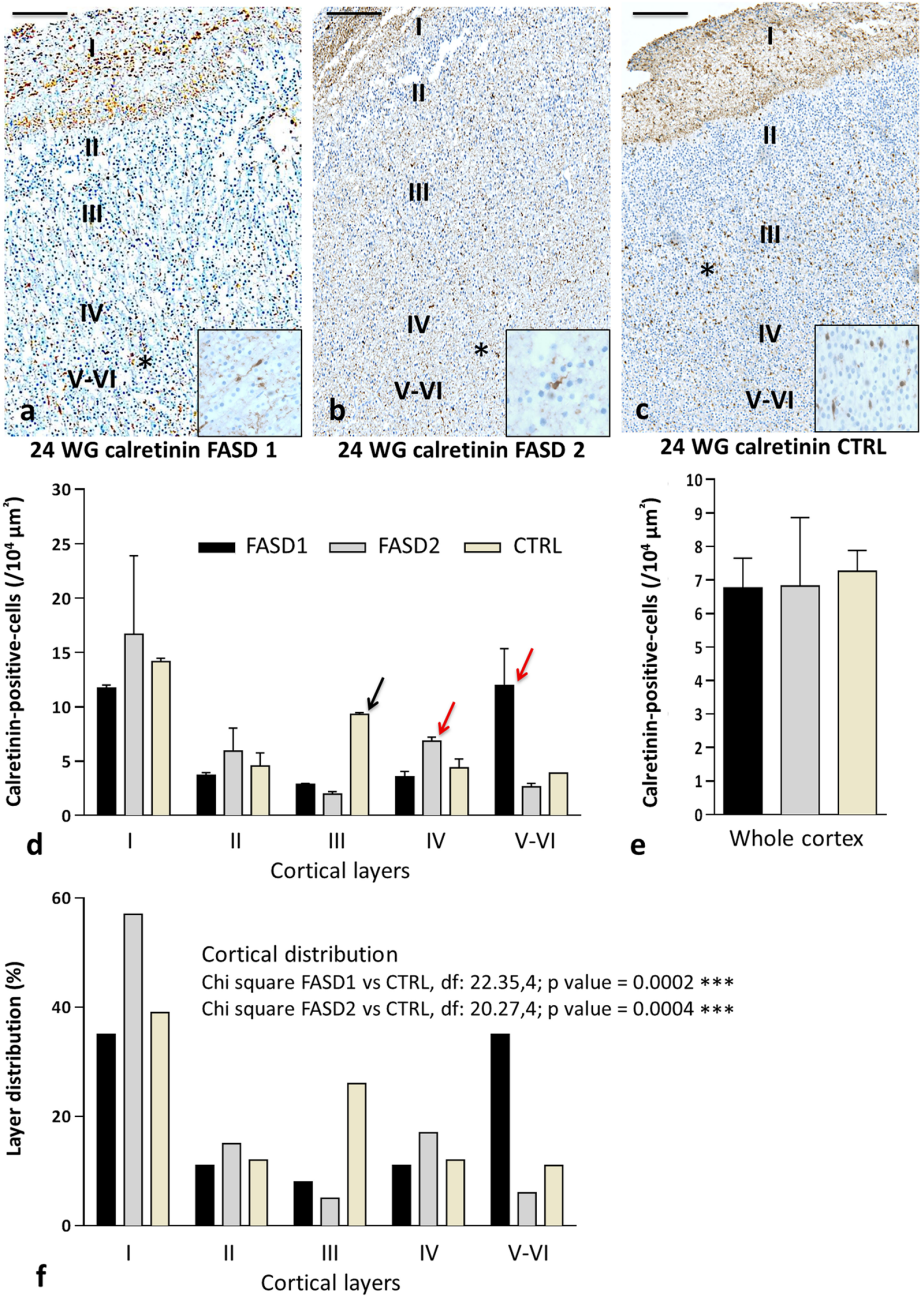
Calretinin immunohistochemistry in cortical plate of FASD and control brains at 24 WG. Random cortical distribution of CR interneurons in FASD brains with a predominance in deeper layers (asterisks) (**a, b**) compared to control cortical plate in which CR cells were more abundant in layer III (asterisk) (**c**). Quantitative analysis of cortical distribution of CR interneurons in each layer showing a predominance in layer III in normal brain (black arrow) and in deeper layers in FASD brains (red arrows) (**d**), associated with a lower global density in FASD brain compared to normal brain (**e**). Intrinsic distributions of CR-positive cells were compared in cortical layers I-VI from the control fetus and the FASD cases. Inserts within Fig. 3a, b ad c illustrate the bipolar morphology of CR interneurons at an original magnification of 400. Chi square analysis indicated that the cortical distributions significantly differed between the control case and both FASD cases (f). Scale bar = 0,18 mm

**Fig. 4:**
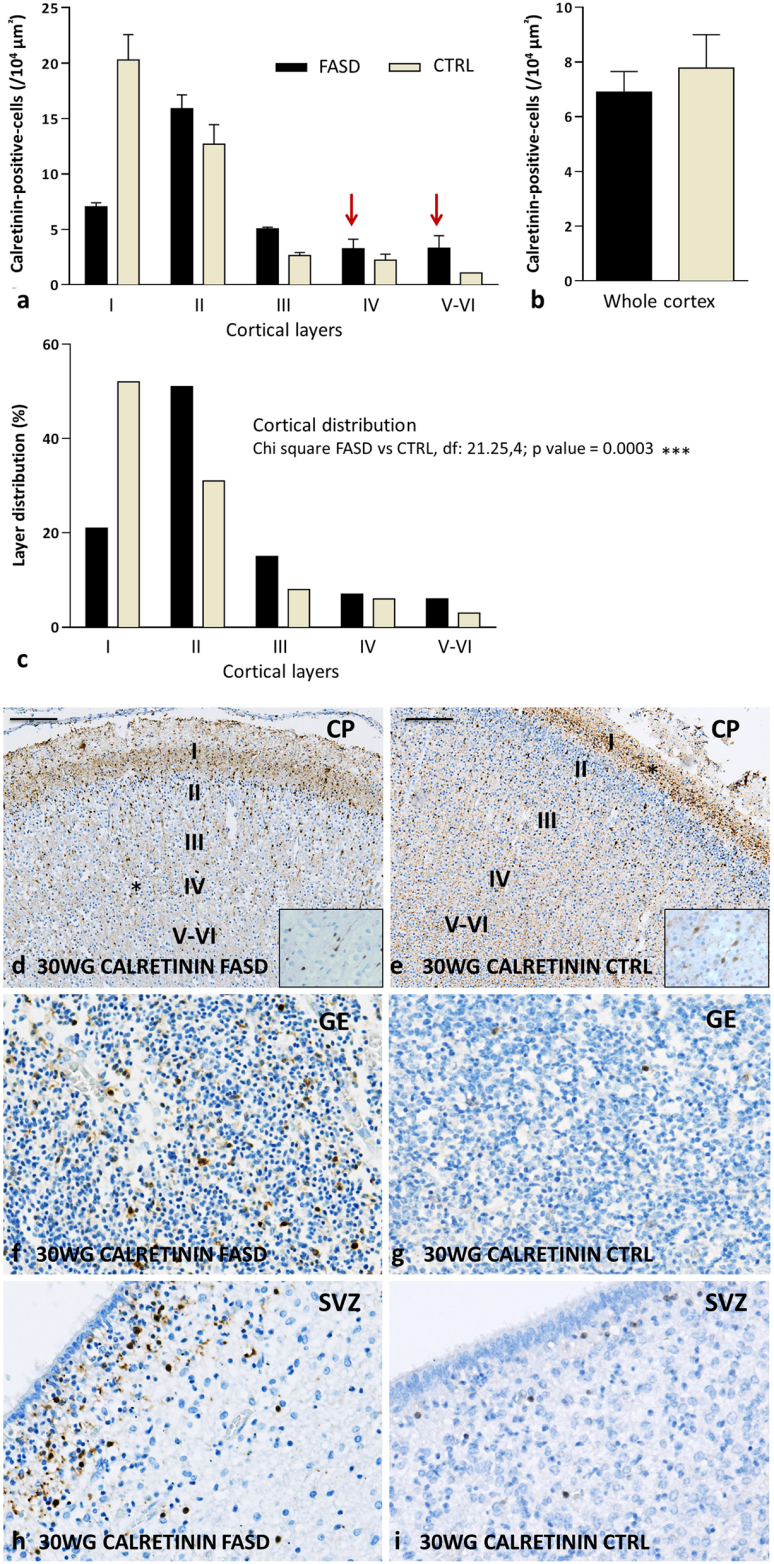
Calretinin immunohistochemistry in the brains of FASD and control at 30 WG. Quantitative analysis of cortical distribution of CR interneurons in each layer showing a predominance in superficial layers in particular layer I in normal brain whereas they were still present in the deeper layers in FASD brain (red arrows) (**a**), associated with a lower global density in FASD brain compared to normal brain (**b**). Statistical analysis using Chi square again confirmed that the cortical distributions significantly differed between the control and the FASD cases (c). Cortical distribution of CR interneurons which were still present in the deeper layers in FASD brain (asterisk) (**d**) compared to control cortical plate in which CR cells were located in superficial layers, in particular layer I (asterisk) (**e**). CR interneurons were more abundant respectively in GE (**f, g**) and SVZ (**h, i**) of the FASD brain compared to control [OM x 20]. Scale bar = 0,18 mm. Inserts within Fig. 4d and e illustrate the bipolar morphology of CR interneurons at an original magnification of 400. CP: cortical plate; CTRL: control cases; GE: ganglionic eminence; FASD: foetal alcohol spectrum disorder; OM: original magnification; SVZ: cortical subventricular zone

**Fig. 5:**
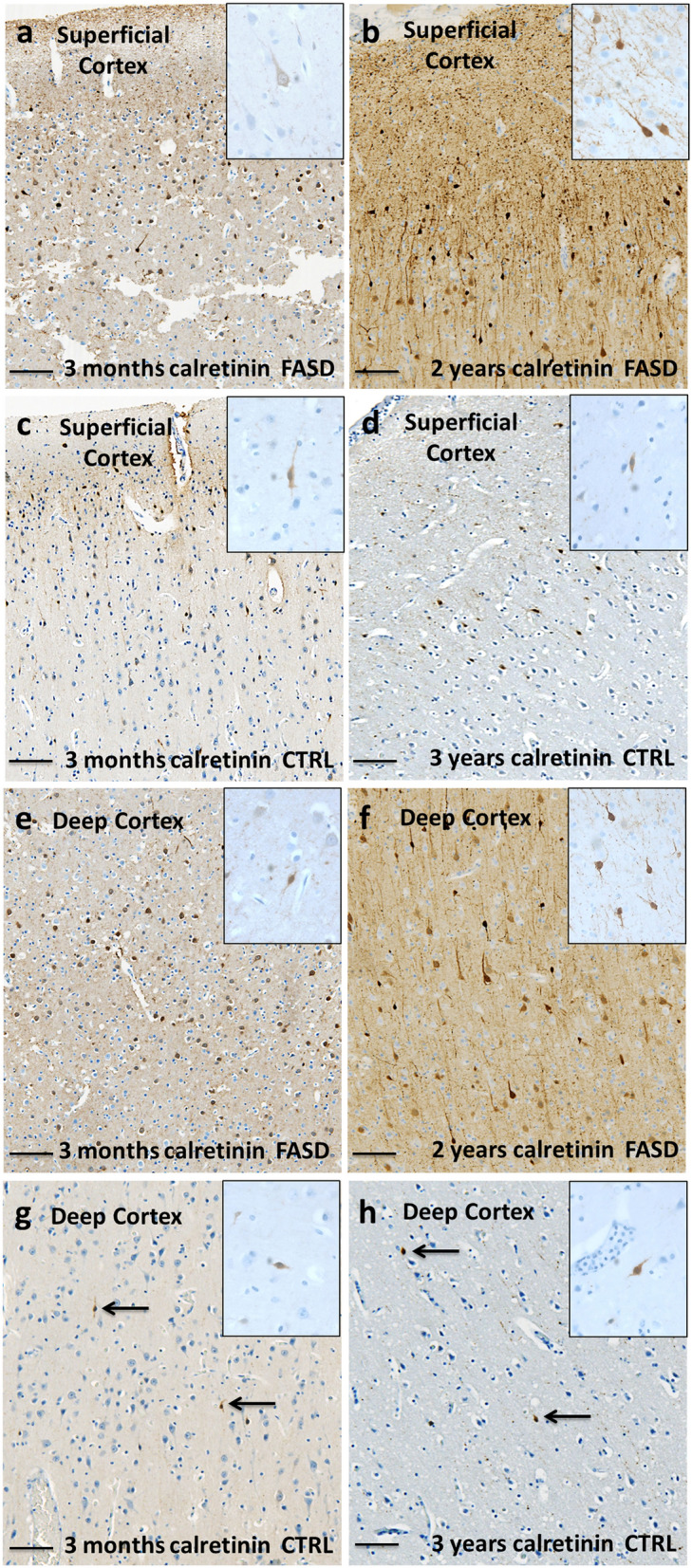
Calretinin immunohistochemistry in the cortex of FASD and control brains after birth. CR cells were more abundant in superficial and especially in deeper cortical layers of FASD brains at 3 months and 2 years (**a** and **b**, **e** and **f**) in comparison with control brains (**c** and **d, g** and **h**). Scale bar = 0,22 mm. Inserts within Fig. 5 illustrate the bipolar morphology of CR interneurons and their neuritic extensions at an original magnification of 400

In the cortical plate, CR-positive INs were observed in all layers from 14 WG and between 14 and 16 WG, and no obvious differences were observed between FASD and controls in the GE, cortical VZ/SVZ and cortical plate. But whereas a significant production in the GE and migration of CR INs in the cortical VZ/SVZ was observed from 18 WG in controls, they were less numerous in the cortical plate (Fig. 3a–c), VZ/SVZ and GE of FASD brains at 20, 22 WG and 24 WG. Quantitative analysis of CR INs performed on two FASD foetal brains at 24 WG confirmed that CR-positive cell density was lower in the cortical plate. It also revealed mispositioning of these cells which were haphazardly located within the different layers and predominated in the deepest layers instead of being mainly located in the upper layers of the cortical plate (Fig. 3d, e). A Chi square comparison of the intrinsic distribution of CR-positive cells in cortical plate showed that the two 24 WG FASD brains significantly differed (Chi^2^ = 22.35 and 20.27, df 4, *p* < 0.0005***) from the control brain (Fig. 3f). These results were further confirmed at 30 WG, i.e., lower global cell density in the cortical plate, still predominating in the deep layers of the cortical plate in case of FASD (Fig. 4a–e), with a significant difference between FASD and control (Fig. 4c, Chi^2^ = 21.25, df 4, *p* < 0.0005***). Besides, from 26 WG the production of CR INs also followed that of GABA in the germinal zones except for two small peaks of production observed at 26 and 30 WG in the GE and VZ/SVZ of FASD brains (Fig. 4f–i). Similar anomalies were identified in the cortical plate of the FASD brain at 37 WG compared to 5 control brains aged 35–39 WG. In the 2 FASD post-natal cases, CR INs were still misplaced in the deepest cortical layers and appeared more numerous than in the cortex of age-matched controls (Fig. 5). Since differences between FASD and control post-natal brains were obvious, quantitative and statistical analyses were not performed in these cases.

As for GABA, semi-quantitative analyses are detailed in Additional file 3 and summarized in Fig. 2. Detailed results obtained from quantitative analyses are presented in Additional file 4.

### Confocal analyses of vascular interneuron migration in the cortical plate

**Fig. 6:**
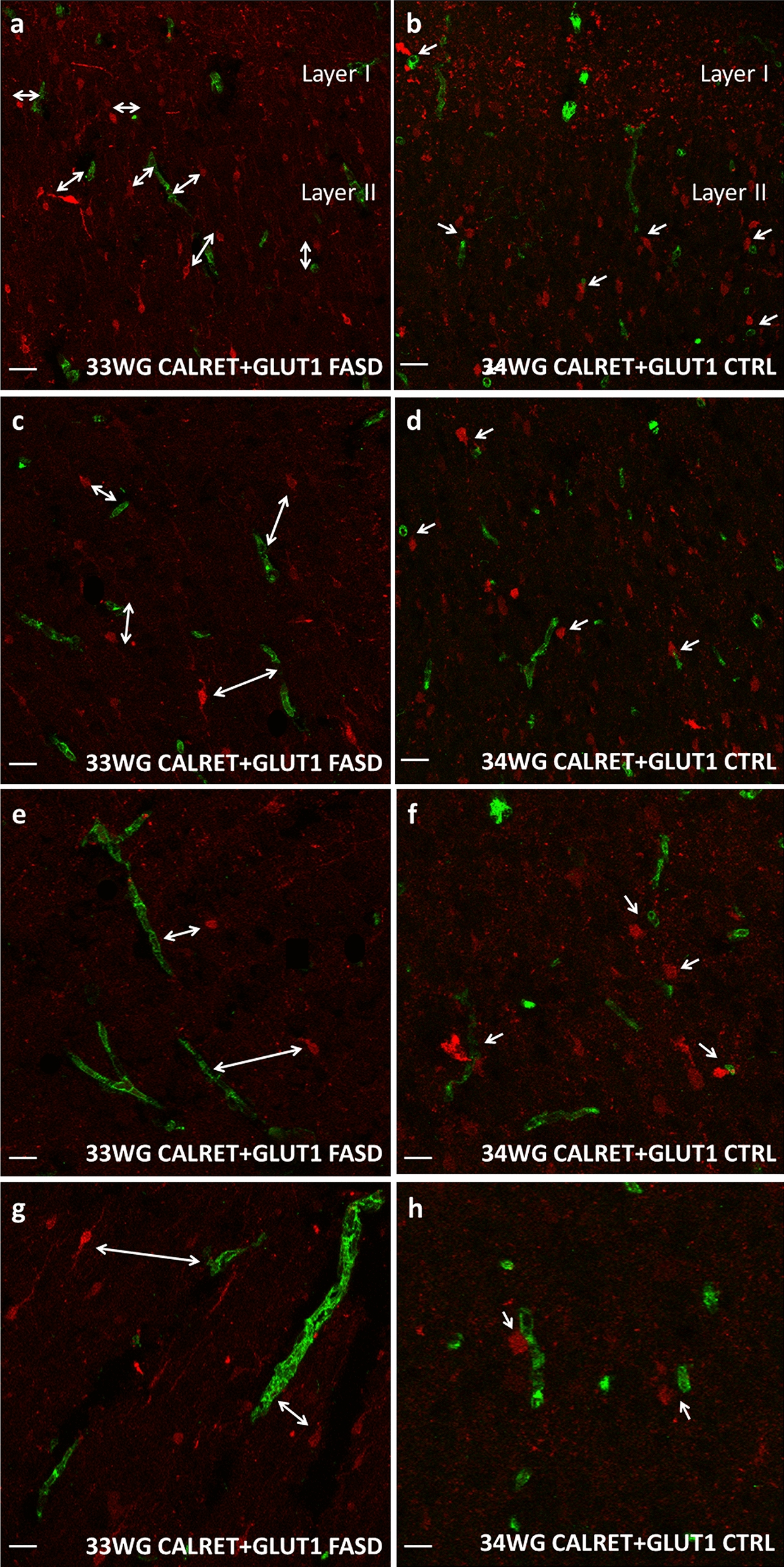
Confocal analysis with calretinin and GLUT1 immunolabellings at 33-34 WG. CR-immunoreactive interneurons, labeled in red, remaining at a distance from the vessels, labeled in green (double-headed arrows) in FASD brain (**a, c, e, g**) contrary to control, where CR-positive interneurons were found in close contact with the vessels (arrows) in all cortical layers (**b, d, f, h**). Scale bar = 5 μm

Close to the pia, we also observed CR-positive neurons on the surface of the cerebral hemispheres, which correspond to the migration of GABAergic INs from the GE to the olfactory bulb via the rostral migratory stream, then to the marginal zone to form the SGL also named Brun layer. About 50% of these cells, which are known to migrate into the underlying cortex, were calretinin-immunoreactive and were observed both in FASD and control brains until 26 WG [41]. Before mid-gestation, no migratory abnormalities of calretininergic INs along the perforating microvessels of the cortical plate were identified. But at later stages, in particular between 33 and 37 WG, vascular migration was affected in the cortical plate of FASD foetuses, in which the majority of CR-immunoreactive INs remained at a distance from the vessels, contrary to controls, where CR-positive INs were found in close contact with the vessels in all layers (Fig. 6 and Additional file 5). When counting on one field from the confocal images including all cortical layers at 34 WG, at a magnification of 400, evident differences concerning the connection between INs and vessels were noted. In the foetus exposed to alcohol, a total of 16 INs were located at a distance of the vessel walls and only 2 were in contact with them, conversely to the control in which 18 INs were in close contact with the vessel walls. Moreover, as previously reported [25], the physiological predominant radial organization of the vasculature was markedly altered.

## Discussion

This study allows to confirm that GABA and CR interneurons are generated early during the foetal period with an intense interneuron generation within the GE from 18 WG followed by a progressive extinction by 30 WG, consistent with previous studies [59, 69]. Interestingly, the CaBP marker CR displayed a dynamic expression, as it reached a peak between 20 and 38 WG and decreased afterwards, suggesting that transient expression of CR-positive neurons could exert precise functions during specific developmental events, in particular during neuronal migration [60]. The main hallmarks of FASD interneuronopathy consisted in a delayed generation of GABA INs expressing calretinin which started from 24 WG instead of 18 WG and resulted in an inadequate number of INs, i.e., insufficient in the cortical plate until birth, in excess after birth, as well as in a failure to integrate themselves at their appropriate location within the different layers according to an inside-out pattern. These anomalies in cell density were always associated with a mispositioning of calretinin INs located in the deepest layers in FASD whereas they were mainly located in the superficial layers in controls, as previously described [48]. It has been postulated that in humans, the location of CR INs in the upper cortical layers (II/III) might play a role in cortical circuit formation necessary for higher brain functions, notably abstract thinking and language [48] which could partly explain the neurocognitive and behavioural deficits observed in FADS. Interestingly, recent studies demonstrated that in a mouse model of monointoxication, PAE affected the expression of some genes involved in intraneocortical connectivity establishment, notably *Rzrβ*, *Cad8* and *Id2* the promoter of which being hypomethylated concomitantly to global DNA hypomethylation that further emphasizes the role of alcohol in altering epigenetic programs [1].

To our knowledge, no study concerning the effects of alcohol during human or rodent brain development has focused on GE. Nevertheless, studies on the effects of alcohol on VZ/SVZ of the dorsal telencephalon in a murine model of cultured radial glial cells and in murine embryonic cerebral cortices revealed that in utero ethanol exposure impairs cell proliferation and results in a decreased production of neurons and astrocytes [52]. Telencephalic cultures obtained from ethanol-treated rats displayed a reduction of actively dividing radial glia progenitors, and neurosphere formation assay showed a reduced number of multipotent progenitor cells in cultures isolated from ethanol-treated rats. More recently, using in vivo and in vitro mouse models, it has been demonstrated that alcohol hinders basal progenitor proliferation in the SVZ by interfering with the cell cycle at G1-S transition from early development [49]. From these observations, it might be suggested that a similar mechanism occurs in the GE, which could explain defective/delayed production of GABAergic INs during foetal life and even microcephaly due to alcohol-induced teratogenic effects on germinative zones. Brain growth failure which is the most frequent brain structural anomaly could also be related to alcohol-induced neuroapoptosis which was first reported 20 years ago by Ikonomidou et al. who showed that the proapoptotic effects of alcohol mediated by both extrinsic and intrinsic pathways were mimicked by NMDA blockers and GABA_a_ receptor modulators and peaked at P7 in rats, which corresponds to 34-38 WG in humans [23]. Since then, it has been shown that nearly all types of neurons including INs are affected by this process.

Several studies have suggested that in rodents and monkeys, the defects in the number of GABA cortical IN and in their positioning in the cortex of FADS patients resulted from impaired migration but data remain contradictory and this could be partly due to the plethora of alcohol exposure schedules and animal models used. Upon alcohol exposure, fewer GABA INs and more particularly in layers II and III have been reported in the somato-sensory cortex of guinea pigs and monkeys [6, 42] suggesting that late born interneurons are susceptible to alcohol exposure. Conversely, other studies reported an increased density of GABA INs in mouse prefrontal cortex, which was attributed to enhanced tangential migration [13, 56]. It is recognized that tangential migration is largely controlled by GABA signaling and that a reduction in ambient GABA results in improper migration of GABA INs which express GABA_a_, GABA_b_ and GABA_c_ receptors and are a target of alcohol [55]. On the other hand, alcohol has been shown to potentiate GABA-mediated signaling by increasing GABA release and GABA_a_ receptor activity with subsequent premature migration. According to Skorput et al., INs of layer V are particularly sensitive to alcohol which could explain increased migration to this layer [56]. Otherwise, IN migration depends on neurotrophic factor activity which regulates the tangential migration mode, such as BDNF, GDNF and HGF which play a crucial role in the dispersion and appropriate location of INs arising from the GE in the dorsal telencephalon [47]. Basic fibroblast growth factor stimulates the generation and differentiation of CR INs, and its effects are enhanced by retinoic acid which plays an essential role in stem cell differentiation and development and is a major target of alcohol [43, 54].

Migration and positioning of GABAergic INs in the cortex is also controlled by cortical microvessels [65] and it has previously been demonstrated that cortical angiogenesis in foetal human brains is impaired by antenatal alcohol exposure [25, 29]. In the present study, we observed that a significant proportion of GABAergic/CR INs were located at a distance from cortical vessels from the beginning of the third trimester of gestation which could also explain IN mispositioning in FASD brains. Recently, *Léger* et al. reported that during normal neurodevelopment, glutamate stimulates activity of the endothelial proteases MMP-9 and t-PA along the pial and radial cortical microvessels. They showed that t-PA invalidation and in vivo administration of a MMP inhibitor resulted in a mispositioning of GABA INs which were missing in the superficial cortical layers supporting the fact that glutamate, via its t-PA-dependent endothelial NMDA receptor, controls vessel-associated migration of GABA INs by regulating endothelial protease activity [30]. Furthermore, these authors provided for the first time mechanistic and functional evidence that upon in utero exposure, alcohol impairs glutamate-regulated activity of pial microvessels and IN positioning by altering metalloproteinase activity. From these results, it could be suggested that alcohol-induced endothelial dysfunction may contribute to ectopic cortical GABAergic IN positioning observed in the present FASD cases [31].

## Conclusion

This study provides further evidence that alcohol affects the GABAergic system in humans from early foetal life by impacting on critical stages of brain development and thus inducing an interneuronopathy. Semi-quantitative and quantitative analyses of GABAergic and calretininergic interneuron density allowed us to identify an insufficient and delayed production of GABAergic interneurons in the ganglionic eminences during the two first trimesters of the pregnancy, a delayed incorporation into the laminar structures of the frontal cortical plate and a mispositioning of GABAergic and calretininergic interneurons which persisted throughout the foetal life. Moreover, vascular migration of calretininergic interneurons within the cortical plate appeared abnormal, as reflected by low numbers of interneurons observed close to the cortical perforating vessel walls that may in part explain their abnormal intracortical distribution. Our results are globally concordant with those previously obtained in mouse and in vitro models, in which alcohol has been shown to induce an interneuronopathy by affecting interneuron density and positioning within the cortical plate, and which could account for the cognitive and behavioral disabilities as well as epilepsy observed in children with foetal alcohol disorder spectrum.

## Supplementary information

**Additional file 1: Table 1.** Semi-quantitative analysis of immunohistochemical data with GABA antibody.

**Additional file 2:** Quantitative analysis of GABA immunolabelling in each layers and in whole cortex (Calretinin-positive-cells/10^4^ µm^2^).

**Additional file 3: Table 2.** Semi-quantitative analysis of immunohistochemical data with Calretinin antibody.

**Additional file 4:** Quantitative analysis of Calretinin immunolabelling in each layers and in whole cortex (Calretinin-positive-cells/10^4^ µm^2^).

**Additional file 5: Figure 1.** Confocal analysis with calretinin and GLUT1 immunolabellings at 36-37 WG. The network of CR fibers (asterisk), labeled in red, was observed in layer I but no CR interneuron was observed in the superficial layers of FASD cortex at 37 WG (**a**) while they were abundant and migrated along the vessels, labeled in green, in superficial layers of control cortex at 36 WG (arrows) (**b**). In another superficial area, CR interneurons were at a distance from the vessels (double-headed arrows) in the FASD brain (**c**) while they were always more abundant and in close contact with the vessels in control (arrows) (**d**). In deeper layers, CR interneurons were more abundant in FASD cortex but at a distance from the vessels (double-headed arrows) (**e**), in contrast with control where they were less abundant and still in close contact with the vessels (arrows) (**f**). Scale bar = 10 μm.

## Abbreviations

CGE: Caudal ganglionic eminence
CNS: Central nervous system
CP: Cortical plate
CR: Calretinin
FAS: Foetal alcohol syndrome
FASD: Foetal alcohol spectrum disorder
GABA: γ-aminobutiric acid
GE: Ganglionic eminences
IN: Interneuron
IUFD: *In Utero* foetal death
IUGR: Intra uterine growth retardation
LGE: Lateral ganglionic eminence
MGE: Median ganglionic eminence
ROI: Regions of interest
SGL: Subpial transient Granular cell Layer
SVZ: Subventricular zone
TOP: Medical termination of the pregnancy
VZ: Ventricular zone
WG: Weeks’ gestation

## Availability of data and materials

Most data generated or analyzed during this study are included in this article. Additional datasets are available from the corresponding author on request.
