# Supplementary material for: Prenatal alcohol exposure is a leading cause of interneuronopathy in humans
Source: Acta Neuropathol Commun. 2020 Nov 30;8:208. doi: 10.1186/s40478-020-01089-z (PMC7706035; doi:10.1186/s40478-020-01089-z)
Supplement: Supplementary file 1 — Additional file 1: Table 1. Semi-quantitative analysis of immunohistochemical data with GABA antibody. [file 40478_2020_1089_MOESM1_ESM.doc]

**Supplementary Table 1**: Semi-quantitative analysis of immunohistochemical data with GABA antibody

| **Term** | **FASD/CTRL** | **VZ/SVZ** | **GE** | **IZ** | **Subplate** | **CP** |
| --- | --- | --- | --- | --- | --- | --- |
| 15 WG | FASD | 1% + | 5% + | 1% + | <1% | 10% + |
| 14 WG | CTRL 1 | 10% + | >50% ++++ | 10% + | 10% + | 10% + |
| 16 WG | CTRL 2 | 10% + | >50% ++++ | 0 | 1% + | 10% + |
| 20 WG | FASD | 5% + | 50% +++ | 5% + | <5% + | 5% + |
|  | CTRL | 70% ++++ | 80% ++++ | 5% + | 10% + | 20% ++ |
| 22 WG | FASD 1 | 10% + | 20% ++ | 0 | 0 | 5% + |
|  | FASD 2 | 5% + | 10% + | 0 | 0 | 5% + |
|  | FASD 3 | 5% + | 5% + | 0 | 0 | 10% + |
|  | CTRL | 60% ++++ | 70% ++++ | 20% ++ | NA | 15% ++ |
| 24 WG | FASD 1 | 20% ++ | 10% + | <5% + | NA | 10% + |
|  | FASD 2 | 20% ++ | 30% ++ | <5% + | NA | 10% + |
|  | CTRL | 60% ++++ | >50% ++++ | 10% ++ | NA | 20% ++ |
| 26 WG | FASD 1 | 5% + | 15% ++ | 10% + | dispersed | 20% ++ |
|  | FASD 2 | 5% + | 5% + | 20% ++ | dispersed | 20% ++ |
|  | CTRL | 10% + | 5% + | <5% + | NA | 15% ++ |
| 29 WG | FASD | 5% + | 1% + | 10% + | NA | 20% ++ |
| 28 WG | CTRL | 0 | 10% + | 20% ++ | scant | 22% ++ |
| 30 WG | FASD | 5% + | 10% + | 10% ++ | NA | 23% ++ |
|  | CTRL | 12% ++ | 5% + | 10% ++ | 10% ++ | 24% ++ |
| 31 WG | FASD 1 | NA | NA | 10% + | NA | 30% +++ |
|  | FASD 2 | TF | TF | TF | TF | TF |
| 32 WG | CTRL | 10% + | 10% + | 20% ++ | scant | 25% ++ |
| 33 WG | FASD | NA | NA | 10% + | NA | 31% +++ |
| 34 WG | CTRL | 5% + | 10% + | 20% ++ | NA | 28% +++ |
| 37 WG | FASD | NA | NA | NA | NA | 32% +++ |
| 35 WG | CTRL 1 | NA | NA | NA | NA | NA |
| 36 WG | CTRL 2 | NA | NA | NA | NA | NA |
| 36 WG | CTRL 3 | 3% + | NA | numerous | NA | 34% +++ |
| 37 WG | CTRL 4 | NA | NA | NA | NA | NA |
| 39 WG | CTRL 5 | NA | NA | NA | NA | NA |
|  |  |  |  |  |  |  |
| 3 months | FASD | absent | absent | 10% + | NA | 38% +++ |
|  | CTRL | absent | absent | 10% + | NA | 35% +++ |
| 2 years | FASD | absent | absent | dispersed | NA | 38% +++ |
|  | CTRL | absent | absent | dispersed | 10% + | 35% +++ |

CP: cortical plate; CTRL: control; FASD: fetal alcohol spectrum disorder ; GE: ganglionic eminences; IZ: intermediate zone; NA: not available ; TF: technical failure ; VZ/SVZ: cortical ventricular and subventricular zones
