# Supplementary material for: Prenatal alcohol exposure is a leading cause of interneuronopathy in humans
Source: Acta Neuropathol Commun. 2020 Nov 30;8:208. doi: 10.1186/s40478-020-01089-z (PMC7706035; doi:10.1186/s40478-020-01089-z)

**Supplementary material 1:** Quantitative analysis of GABA immunolabelling in each layers and in whole cortex (Calretinin-positive-cells/10^4^ µm²)

**At 26WG :**


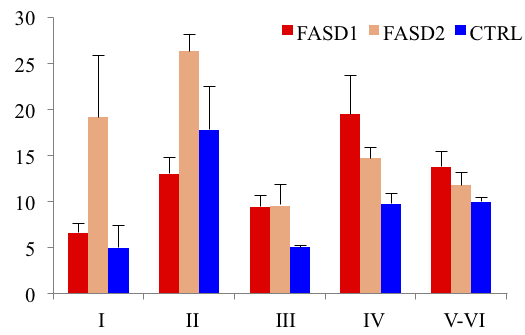

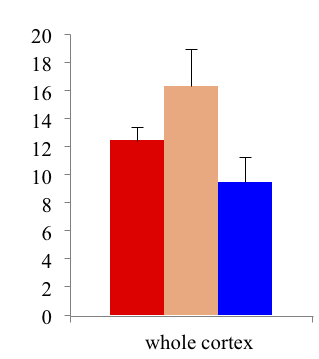


**At 28-29WG:**


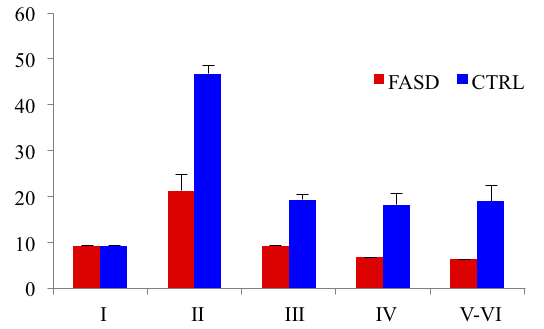

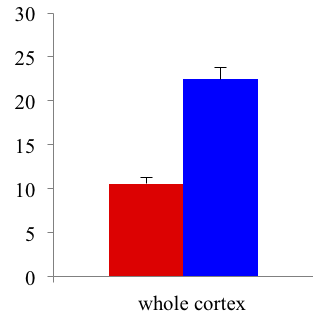


**At 30WG:**


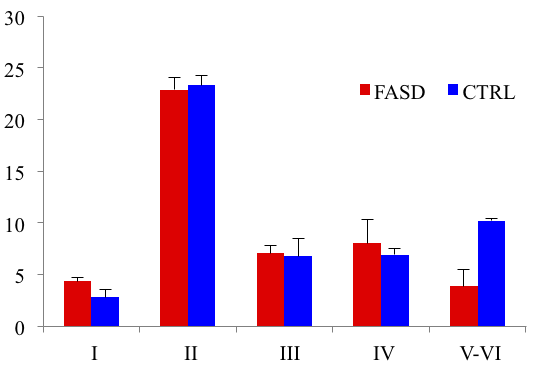

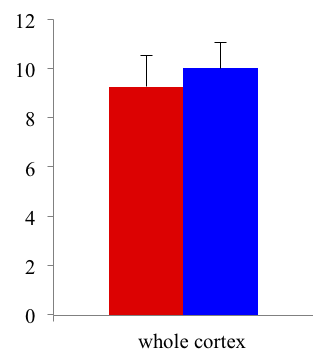


**At 31-32WG:**


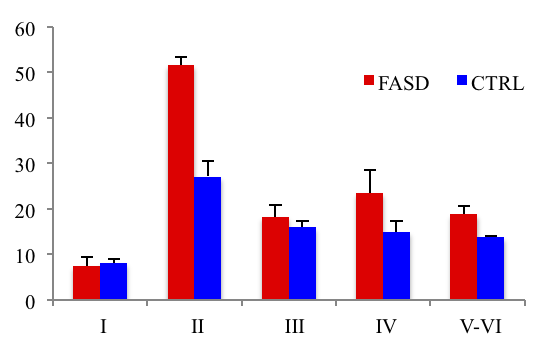

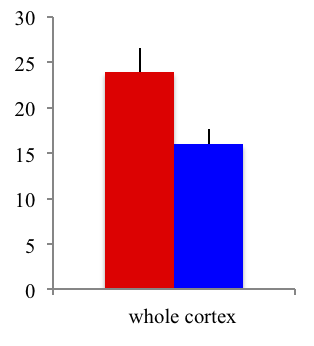


**At 33-34WG:**


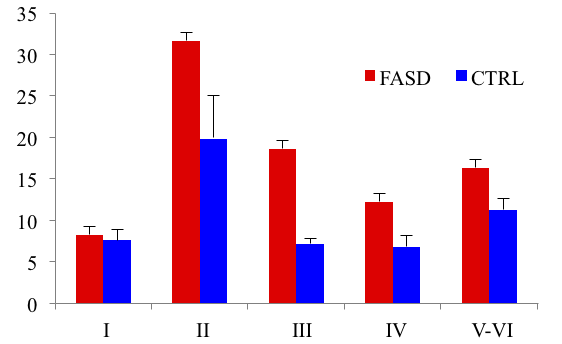

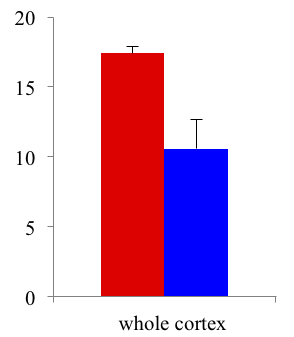


**At 36-37WG:**


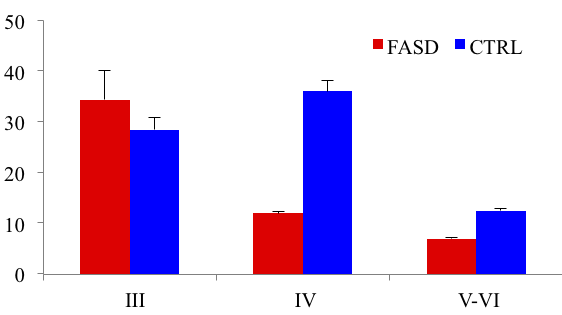

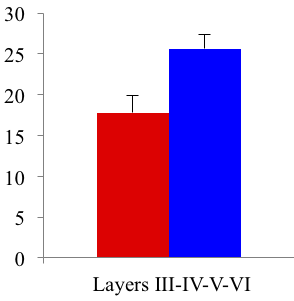


**At 3 months after the birth:**


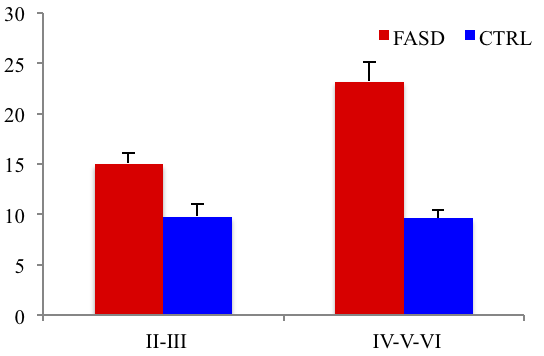

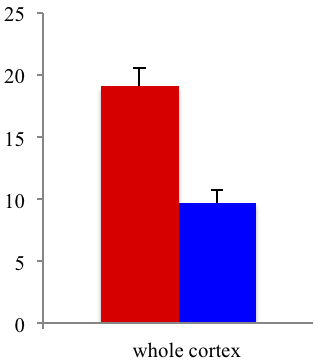

Supplement: Supplementary file 2 — Additional file 2: Quantitative analysis of GABA immunolabelling in each layers and in whole cortex (Calretinin-positive-cells/104 µm2). [file 40478_2020_1089_MOESM2_ESM.doc]
