# Supplementary material for: Prenatal alcohol exposure is a leading cause of interneuronopathy in humans
Source: Acta Neuropathol Commun. 2020 Nov 30;8:208. doi: 10.1186/s40478-020-01089-z (PMC7706035; doi:10.1186/s40478-020-01089-z)
Supplement: Supplementary file 3 — Additional file 3: Table 2. Semi-quantitative analysis of immunohistochemical data with Calretinin antibody. [file 40478_2020_1089_MOESM3_ESM.doc]

**Supplementary Table 2**: Semi-quantitative analysis of immunohistochemical data with Calretinin antibody

| **Term** | **FASD/CTRL** | **VZ/SVZ** | **EG** | **IZ** | **subplate** | **CP** |
| --- | --- | --- | --- | --- | --- | --- |
| 15 WG | FASD | 5-10% + | 5% + | 5% + | 5% + | 2% + (dispersed) |
| 14 WG | CTRL 1 | 5% + | 5% + | 5% + | 5% + | 7% + (dispersed) |
| 16 WG | CTRL 2 | 5-10% + | 5-10% + | 1% + | 1% + | 7% + (dispersed) |
| 20 WG | FASD | 5% + | 5% + | 0 | 0 | 2% + (dispersed) |
|  | CTRL | 5-10% + | 15% ++ | 5% + | 5% + | 15% ++ (dispersed) |
| 22 WG | FASD 1 | 10% + | 5-10% + | <10% + | NA | 10% + (dispersed) |
|  | FASD 2 | 5% + | 5% + | 1% + | 0 | 2% + (dispersed) |
|  | FASD 3 | <5% + | <5% + | 1% + | NA | 2% + (dispersed) |
|  | CTRL | 30% +++ | 30% +++ | 20% ++ | NA | 20% ++ (layers IV-V-VI) |
| 24 WG | FASD 1 | 5-10% + | 5% + | 10-20% ++ | 10% + | 15% ++ (dispersed) |
|  | FASD 2 | 5% + | 5% + | 5% | NA | 15% ++ (dispersed) |
|  | CTRL | 10% + | 10% + | 10% + | NA | 20% ++ (deep layer III) |
| 26 WG | FASD 1 | 5-10% + | 15% ++ | 5% + | <5 % + | 15% ++ (dispersed) |
|  | FASD 2 | 5% + | 5% + | scant | <1% + | 15% ++ (dispersed) |
|  | CTRL | 5% + | 5% + | 5-10% + | 5% + | 20% ++ (superficial layer III) |
| 29 WG | FASD | <1% + | <1% + | scant | 0 | 15% ++ (dispersed) |
| 28 WG | CTRL | 0 | <1% + | 5% + | NA | 20% ++ (layers II-III) |
| 30 WG | FASD | 10% + | 10-15% ++ | 5% + | NA | 15% ++ (dispersed) |
|  | CTRL | 5% + | 2% + | 10% + | NA | 20% ++ (layers II-III) |
| 31 WG | FASD 1 | 5% + | 5% + | 5-10% + | 0 | 15% ++ (dispersed) |
|  | FASD 2 | NA | NA | scant | 0 | 15% ++ (dispersed) |
| 32 WG | CTRL | <5% + | 5% + | 5-10% + | NA | 20% ++ (layers II-III) |
| 33 WG | FASD | 5% + | 1% + | <5% + | NA | 20% ++ (dispersed) |
| 34 WG | CTRL | 5% + | 5% + | 10% + | NA | 25% ++ (layers II-III) |
| 37 WG | FASD | NA | NA | 5% | NA | 15% ++ (deep layers) |
| 35 WG | CTRL 1 | NA | NA | NA | NA | 20% ++ (superficial layers) |
| 36 WG | CTRL 2 | NA | NA | NA | NA | 20% ++ (superficial layers) |
|  | CTRL 3 | 1% + | 1% | 5% | NA | 20% ++ (superficial layers) |
| 37 WG | CTRL 4 | NA | NA | NA | NA | 20% ++ (superficial layers) |
| 39 WG | CTRL 5 | NA | NA | NA | NA | 20% ++ (superficial layers) |
|  |  |  |  |  |  |  |
| 3 months | FASD | absent | absent | NA | NA | 20% ++ (layers II-III and V) |
|  | CTRL | absent | absent | NA | NA | 5% + (layers II-III) |
| 2 years | FASD | absent | absent | NA | NA | 20% ++ (layers II-III and V) |
|  | CTRL | absent | absent | NA | NA | 5% + (layers II-III) |

CP: cortical plate; CTRL: control; FASD: fetal alcohol spectrum disorder; GE: ganglionic eminences; IZ: intermediate zone; NA: not available; TF: technical failure; VZ/SVZ: cortical ventricular and subventricular zones
